# Association among Working Hours, Occupational Stress, and Presenteeism among Wage Workers: Results from the Second Korean Working Conditions Survey

**DOI:** 10.1186/2052-4374-26-6

**Published:** 2014-03-24

**Authors:** Sung-Hwan Jeon, Jong-Han Leem, Shin-Goo Park, Yong-Seok Heo, Bum-Joon Lee, So-Hyun Moon, Dal-Young Jung, Hwan-Cheol Kim

**Affiliations:** 1 Department of Occupational and Environment Medicine, School of Medicine, Inha University, Incheon, South Korea

**Keywords:** Presenteeism, Occupational stress, Working hours, Shift work

## Abstract

**Objectives:**

The purpose of the present study was to identify the association between presenteeism and long working hours, shiftwork, and occupational stress using representative national survey data on Korean workers.

**Methods:**

We analyzed data from the second Korean Working Conditions Survey (KWCS), which was conducted in 2010, in which a total of 6,220 wage workers were analyzed. The study population included the economically active population aged above 15 years, and living in the Republic of Korea. We used the chi-squared test and multivariate logistic regression to test the statistical association between presenteeism and working hours, shiftwork, and occupational stress.

**Results:**

Approximately 19% of the workers experienced presenteeism during the previous 12 months. Women had higher rates of presenteeism than men. We found a statistically significant dose–response relationship between working hours and presenteeism. Shift workers had a slightly higher rate of presenteeism than non-shift workers, but the difference was not statistically significant. Occupational stress, such as high job demand, lack of rewards, and inadequate social support, had a significant association with presenteeism.

**Conclusions:**

The present study suggests that long working hours and occupational stress are significantly related to presenteeism.

## Introduction

Workers’ health is not only their own issue, but also an important issue for their employers. The illness of workers can result in lost workforce productivity and add to the disease burden of the company or community. One study found that estimated costs of health-related productivity loss were significantly greater than medical and pharmacy costs [1]. Generally, productivity loss due to workers’ poor health arises from absenteeism or presenteeism. Absenteeism, which is the easily understandable concept of absence from work due to disease, places a well-known economic burden on industry. In contrast, presenteeism is a newer economic concept and has recently been recognized as a health problem in the workplace.

Presenteeism is defined as being present at work, but limited in some aspects of job performance by a health problem [2]. Working while sick can lead to many negative consequences, such as lost productivity, reduced work team cohesion, accidents, job insecurity/turnover, worsening health, and longer recovery time [3]. The economic burden of presenteeism has been overlooked because it is not as obvious to employers or workers as the economic costs due to absenteeism. However, according to the *Harvard Business Review*, the estimated total cost of presenteeism in the United States was more than $150 billion per year, and the economic cost of presenteeism was far more than that of absenteeism or disability [4].

Sickness presenteeism can also be a risk factor for other adverse health events. Sickness absenteeism provides time for a recovery period and appropriate medical management to ill and distressed workers. However, workers who work while sick can not resolve their unhealthy condition and could be faced with a cumulative stress burden. In a recent report based on a cohort of male British civil servants, unhealthy employees who took no absence during a 3-year follow-up had a 2-fold higher incidence rate of serious coronary events than unhealthy employees with a moderate level of sickness absenteeism [5]. Several other studies have also suggested that sickness presenteeism predicts poor self-rated health and future sickness absence [6-8].

The risk factors for presenteeism include both physical and psychological health conditions [1,9-11]. According to a prospective study of about employees of a financial services company, 10 health risk factors (cigarette smoking, physical activities, safety belt use, use relaxation medicine, high blood pressure, obesity, life dissatisfaction, job dissatisfaction, perception of physical health, and high stress) were significantly correlated with presenteeism [12].

In general, long working hours, shift work and occupational stress can exacerbate workers’ chronic health conditions. Long working hours, shift work, and occupational stress lead to unhealthy habits, such as smoking, alcohol abuse, lack of physical activity, sleeplessness, poor eating habits, and fewer chances for medical examinations, consequently aggravating physical conditions [13]. Recent research suggesting long working hours is associated with increased BMI and waist circumference [14], poorer lifestyle, higher stress, and lower quality of life [15], high blood pressure [16], and cardiovascular disease [17,18]. Shift work is associated with occurrence of obesity, dyslipidemia, and metabolic syndrome [19,20], and a higher risk for common infections [21]. Shift work can act as an oxidative stressor and may induce many medical disorders [22]. Association between occupational stress and adverse health outcomes was also supported by many studies. Occupational stress is associated with migraine [23], high blood pressure [24] and coronary heart disease [5,25,26], psychiatric disorders [27-30], such as depression and anxiety. Other research has suggested the association of occupational stress and unhealthy behaviours [31,32], such as smoking and obesity.

Therefore, we hypothesize that those work-related health risk factors (long working hours, shift work, and occupational stress) have a significant association with presenteeism. In fact, some studies suggesting an association between work-related hazardous factors and presenteeism have been conducted [33,34], but no previous investigation has used large-scale representative data from a Korean population.

Hence, the aim of this study was to identify the association between presenteeism and long working hours, shiftwork, and occupational stress using large-scale representative data from the second Korean Working Conditions Survey (KWCS).

## Materials and methods

### Study population

We analyzed data from the second KWCS, which was conducted in 2010, to identify the statistical association between working conditions (occupational stress, work hours, and shift work) and presenteeism. The aim of the KWCS was to identify rates and causes of work-related diseases and accidents, and to verify the effect of mechanical, physical, and chemical hazards in the workplace and psychosocial factors that influence working conditions. The population of the KWCS included the economically active population of those 15 years of age and above who live in Korea. 10,019 participants responded to the questionnaire. We restricted the population of this study to wage workers, and thus 6,220 participants were included. The study population was weighted by the size of a family based on 2011 census of Korea (sum of weights?=?7,112).

### Variables

#### General characteristics

To identify the influence of the study population’s general characteristics on presenteeism, we obtained information regarding demographic and behavioral characteristics (age, sex, educational status, smoking status, alcohol consumption, obesity, depressive symptoms, and history of hypertension) and work-related characteristics (employment status, job type, number of employees, tenure). We classified smoking status into non-smoker, ex-smoker, and current smoker, and alcohol consumption into non-drinker, once per week or less, and twice or more per week. The job types were classified into three categories: white collar, blue collar, and service workers.

#### Workplace environmental risk exposure

We divided workplace environmental risk exposure into three categories: (i) physical risk exposure (vibration, noise, high temperature, low temperature, mist, dust, fumes), (ii) biological/chemical risk exposure (organic solvents, chemical agents, secondhand smoke, infectious agents), and (iii) ergonomic risk exposure (painful or tiring positions, repetitive hand or arm movements, moving or lifting people, heavy loads, standing posture). Workplace environmental risk exposures were classified as dichotomous variables (exposed or unexposed). The population exposed to physical, biological/chemical, and ergonomic risks was defined as those who were exposed to at least one of sub-factors of each of the environmental risk factors during 1/4 or more of their working time.

#### Working hours and shiftwork

Working hours were identified using the following question: “How many hours do you work in a week in your workplace (excluding commuting and meal time)?” Working hours were rounded off to the nearest hour. According to the Korean Labor Standards Act, working hours per week shall not exceed 40 hours excluding break times. If the parties concerned reach agreement, the working hours may be extended by up to twelve hours per week [35]. According to the Enforcement Decree of the Industrial Accident Compensation Insurance Act of Republic of Korea, chronic overwork was defined as exceeding 6 working hours per week for 3 months. Therefore, we categorized working hours as (i) ≤40 hours, (ii) 41-52 hours, (iii) 53-60 hours, and (iv) >60 hours. We defined non-shift workers as those who usually work during the daytime, and the others were classified as shift workers.

#### Occupational stress

Variables about occupational stress were classified and reduced by using the method from “Secondary analysis of Korean working conditions survey: Causes of absenteeism due to disease in employed women” by Kim JE [36]. To measure occupational stress in the workplace, we used five sections: (i) high job demand, (ii) insufficient job control, (iii) inadequate social support, (iv) job insecurity, and (v) lack of rewards. Seven items (rapid speed of work, strict deadlines, interruption of work due to unexpected new tasks, strict standard of quality, self-assessment of work, self-problem solving of unexpected events, insufficient time to work) were used to evaluate high job demand. Seven items (able to spend time handling private or familial tasks during business hours, able to choose or change the order of tasks, able to choose or change methods of work, able to choose or change the speed of work, influence over choice of working partners, able to take a break when desired, influence over making important decisions in tasks) were used to evaluate insufficient job control. Eight items (social support of coworkers, social support of superiors, having very good friends at the workplace, feedback on work by superiors, respect for personality, ability to resolve a conflict, ability to plan or organize work, encouragement to join in important decision making) were used to evaluate inadequate social support. Job insecurity was evaluated using the following 2 questions: (i) “I might lose my job in the next 6 months.” and (ii) “If I leave or lose my current job, I can easily find a new job with the same payment.” Lack of rewards was evaluated using the following question: “I am well paid for the work I do.” Each section was converted to a dichotomous variable (high/low) according to the median value. The Cronbach’s α of each section were: (i) 0.639 (high job demand), (ii) 0.680 (insufficient job control), (iii) 0.775 (inadequate social support).

#### Presenteeism

Presenteeism was identified using the following question: “Over the past twelve months, have you been working, even if you were sick?”

### Statistical analysis

The chi-squared test was applied to identify the statistical association between presenteeism and the possible confounding variables. Univariate and multivariate logistic regression were used to test the statistical association of presenteeism and working hours, shiftwork, and occupational stress. We calculated the odds ratios (ORs) and 95% confidence interval (95% CI) in two models: (i) Model I: crude ORs, (ii) Model II: adjusted by general characteristics and workplace environmental risk exposure (age, gender, education, smoking status, hypertension, obesity, depressive symptoms, job type, tenure, and physical, biological/chemical, and ergonomic risk exposure). Variables for general characteristics whose univariate test had a p-value?<?0.05 were defined as confounding variables and included in Model II as covariates. We used Statistical Package for the Social Sciences software (SPSS version 14.0; SPSS, Inc., Chicago, IL, USA) to conduct the statistical analysis.

## Results

### Demographic and work-related characteristics

In this study, there were more male participants (58.8%) than female participants (41.2%). The mean age was 40.53 years, ranging from 15 to 83.

The associations between general characteristics and presenteeism are shown in Table 1. 1,341 workers (18.9%) had experienced presenteeism. The females (22.2%) had a significantly higher experience rate than males (16.5%). There was a high correlation between hypertension (p-value?<?0.001), obesity (p-value?<?0.001), depressive symptoms (p-value?<?0.001), and presenteeism. Service workers (16.5%) had a significantly lower experience rate than white collar workers (19.7%) or blue collar workers (19.1%). New employees (14.8%), who had tenure of less than a year, had a significantly lower experience rate. Regular workers (19.2%) showed a slightly higher rate of presenteeism than temporary workers (18.2%), but there was no statistical significance (p-value?=?0.291). Those who were exposed to physical or biological/chemical risk factors had a significantly higher rate of experiencing presenteeism than the non-exposure group (p-value?<?0.001). Those who were exposed to ergonomic risk factors showed a slightly higher experience rate than the non-exposure group, but the difference was not statistically significant (p-value?=?0.052).

**Table 1:** The relationship between general characteristics, work-related factors, and presenteeism

			**Presenteeism**		
	**N†**	**%**	**n**	**%**	p-value*
Gender					
Female	2928	41.2	649	22.2	<0.001
Male	4184	58.8	692	16.5	
Age					
<30	1481	20.8	219	14.8	<0.001
30-39	2057	28.9	404	19.6	
40-49	1880	26.4	386	20.5	
50-59	1155	16.2	225	19.5	
≥60	539	7.6	107	19.8	
Educational status					
< Middle school	973	13.7	212	21.8	0.004
High school	2966	41.7	512	17.3	
> College	3172	44.6	616	19.4	
Smoking					
non-smoker	3884	54.6	790	20.3	0.002
ex-smoker	697	9.8	114	16.4	
smoker	2531	35.6	436	17.2	
Drinking frequency					
none	1938	27.2	378	19.5	0.473
≤1 per week	2965	41.7	564	19.0	
≥2 per week	2209	31.1	399	18.1	
Hypertension					
No	6622	93.1	1198	18.1	<0.001
Yes	489	6.9	143	29.2	
Obesity					
No	6886	96.8	1254	18.2	<0.001
Yes	226	3.2	87	38.5	
Depressive symptoms					
No	6999	98.4	1272	18.2	<0.001
Yes	112	1.6	68	60.7	
Employment status					
Regular	4568	64.2	878	19.2	0.291
Temporary	2544	35.8	463	18.2	
Job type					
White collar	3081	43.3	606	19.7	0.043
Service	1350	19.0	223	16.5	
Blue collar	2680	37.7	511	19.1	
Number of employees					
< 5	1526	22.3	285	18.7	0.088
5-49	3295	48.4	599	18.2	
50-299	1315	19.3	246	18.7	
≥ 300	689	10.0	157	22.8	
Tenure (yr)					
< 1	1477	20.8	218	14.8	<0.001
1-10	4084	57.4	826	20.2	
≥10	1550	21.8	296	19.1	
Physical risk exposure					
Unexposed	4348	61.1	745	17.1	<0.001
Exposed	2764	38.9	596	21.6	
Biological/chemical risk exposure					
Unexposed	5126	72.1	887	17.3	<0.001
Exposed	1985	27.9	453	22.8	
Ergonomic risk exposure					
Unexposed	1541	21.7	264	17.1	0.052
Exposed	5569	78.3	1076	19.3	

*Based on the chi-squared test.

†All numbers reflect weighted frequencies.

### Working hours, shift work, occupational stress and presenteeism

The univariate analysis of work-related hazardous factors and presenteeism is shown in Table 2. The group who worked >60 hours per week (27.5%) showed the highest rate of presenteeism, followed by those who worked 53–60 hours per week (20.7%), 41–52 hours per week (19.2%), and ≤40 hours per week (16.6%). Shift workers (20.2%) had a slightly higher rate of presenteeism than non-shift workers (18.7%), but the difference was not statistically significant (p-value?=?0.328). Job stressors, such as high job demand, inadequate social support, and lack of reward, had strong associations with presenteeism (p-value?<?0.001, p-value?=?0.046, p-value?<?0.001), but insufficient job control and job insecurity did not have a significant association.

**Table 2:** The relationship between working hours, shiftwork, occupational stress, and presenteeism

			**Presenteeism**		
	**N†**	**%**	**No**	**%**	p-value*
Working Time (hr/wk)					
≤ 40	3438	48.3	148	16.6	<0.001
41-52	2056	28.9	814	19.2	
53-60	1035	14.6	79	20.7	
> 60	582	8.2	299	27.5	
Shift work					
No	6337	89.1	1185	18.7	0.328
Yes	774	10.9	156	20.2	
High job demand					
Low	5332	75.0	854	16.0	<0.001
High	1779	25.0	487	27.4	
Insufficient job control					
Low	4118	57.9	804	19.5	0.085
High	2993	42.1	536	17.9	
Inadequate social support					
Low	3752	52.7	674	18.0	0.046
High	3360	47.3	666	19.8	
Job insecurity					
Low	2963	41.8	573	19.3	0.363
High	4127	58.2	763	18.5	
Lack of reward					
Low	4265	60.1	743	17.4	<0.001
High	2831	39.9	595	21.0	

*Based on the chi-squared test.

†All numbers reflect weighted frequencies.

The multivariate analysis of work-related hazardous factors and presenteeism is shown in Table 3.

**Table 3:** The odds ratios and 95% confidence intervals of working hours, shiftwork, and occupational stress on presenteeism

	Model I*		**Model II†**	
	**OR**	**95% CI**	**OR**	**95% CI**
Working Time (hr/wk)				
≤ 40	1.00		1.00	
41-52	1.19	1.03-1.37	1.19	1.03-1.38
53-60	1.31	1.10-1.56	1.48	1.23-1.78
> 60	1.90	1.55-2.33	2.10	1.69-2.61
Shift work				
No	1.00		1.00	
Yes	1.10	0.91-1.32	1.22	1.00-1.46
Occupational stress				
High job demand				
Low	1.00		1.00	
High	1.98	1.74-2.25	1.82	1.59-2.09
Insufficient job control				
Low	1.00		1.00	
High	0.90	0.80-1.02	0.93	0.82-1.05
Inadequate social support				
Low	1.00		1.00	
High	1.13	1.00-1.27	1.10	0.97-1.26
Job insecurity				
Low	1.00		1.00	
High	0.95	0.84-1.07	0.92	0.81-1.04
Lack of reward				
Low	1.00		1.00	
High	1.26	1.12-1.42	1.23	1.08-1.39

*Crude odds ratio.

†Adjusted for age, gender, education, smoking status, hypertension, obesity, depressive symptoms, job type, tenure, and physical, biological/chemical, and ergonomic risk exposure.

Working for long hours was a significant risk factor of presenteeism in all of the models. Compared with the reference group who worked ≤40 hours per week, the ORs for those working >60 hours, 53–60 hours, and 41–52 hours per week were 1.898 (95% CI 1.549-2.325), 1.306 (95% CI 1.096-1.556), and 1.192 (95% CI 1.034-1.373) in model I; and 2.098 (95% CI 1.686-2.611), 1.480 (95% CI 1.230-1.783), and 1.192 (95% CI 1.030-1.380) in model II, respectively. We also found a dose–response relationship between working hours and presenteeism in all models (p for trend?<?0.001). We were able to identify a statistically significant association between shift work and presenteeism in model II, but model I did not show statistical significance (OR?=?1.098, 95% CI 0.911-1.323 in Model I; OR?=?1.221, 95% CI 1.001-1.461 in Model II).

The results on the associations of presenteeism and occupational stress were significant in three sections. High job demand was the most powerful stressor that induced presenteeism. The ORs for high job demand were 1.977 (95% CI 1.741-2.246) and 1.822 (95% CI 1.588-2.091) in each of the two models, respectively. We also found statistically significant associations between presenteeism and lack of rewards and inadequate social support. However, negative associations with job insecurity and insufficient job control were shown, but they were not statistically significant.

## Discussion

In this study, we identified associations between presenteeism and work-related hazardous factors by using large-scale representative data from the Korean working population. We found a strong association between presenteeism and chronic health conditions, such as a history of hypertension, obesity, and depressive symptoms. This result suggests that sickness presenteeism could be induced by a worker’s chronic health condition. This hypothesis is supported by recent studies. Mental and physical disorders are associated with additional days of absence and reduced qualitative functioning at work [9,11]. Chronic health conditions, such as depression, anxiety, sleep problems, fatigue, obesity, arthritis, headache, and back/neck pain, are significantly related to absenteeism and presenteeism [1,10]. According to our results, workplace environmental risk exposure was significantly related to presenteeism, except ergonomic risk exposure (p-value?=?0.052). There is a lack of research about the association between presenteeism and workplace environmental risk exposure. However, a variety of workplace environmental risks, such as heavy physical work load, ergonomic conditions, and hazardous exposure, have been found to be associated with sicknessabsence [37,38]. Furthermore, workplace environmental risk exposure may induce many medical disorders. Consequently, hazardous exposures in the workplace are likely to be related to presenteeism.

We also found a significant relationship between working hours and presenteeism. In particular, those who worked over 60 hours per week had a 2-fold higher odds ratio than those who worked 40 or less hours per week. Respondents working >60 hours had highest odds ratio, followed by 53–60 hours and 41–52 hours. We conducted a trend analysis and found a statistically significant result (p for trend?<?0.001). This finding suggests a dose–response relationship between working hours and presenteeism. Our finding of significant relationship between working hours and presenteeism can be explained by the adverse health effects of long working hours. Several studies have suggested that long working hours can be a risk for adverse health events. Those include cardiovascular disease [5,25,26], metabolic disease, such as metabolic syndrome [39], hypertension [40], obesity [41], and mental disorders such as sleep disturbance [42], depression [43], and anxiety [44]. Those chronic health conditions are associated with presenteeism [1] and loss of productivity [9]. Moreover, sleep disturbance and fatigue due to long work hours especially increase the risk of injury and accidents on the job [45], and decrease productivity by reducing job performance [46].

Shift work causes disturbances of the normal circadian rhythms; consequently, shift workers suffer from so-called ‘shift-lag’ syndrome, which is characterized by feelings of fatigue, sleepiness, insomnia, disorientation, digestive troubles, irritability, poorer mental agility, and reduced performance efficiency [47]. Consequently, shift work also can be a risk factor of presenteeism. A Finnish study reported a relationship between presenteeism and shift work, but they did not find statistical significance [48]. In this study, shift workers showed a bit higher experience rate of presenteeism than non-shift workers, and we were able to find a significant association in the adjusted model. The Republic of Korea shows higher proportions of shift workers in the service sector [49]. Therefore, our analysis using overall population could not identify an effect in the highly exposed group. We anticipate further research about the influence of shift work on presenteeism within highly exposed populations.

Our study suggests that high job demand was the most important risk factor of presenteeism among the job stressors. Lack of rewards also had a significant association with presenteeism. However, insufficient job control and job insecurity had a negative correlation, but this was not statistically significant. Psychosocial working conditions have a strong relationship with workers’ general health condition and productivity. Job stressors such as job demand, interpersonal conflict, and lack of reward are correlated with absence and early leave from work [50]. Workers experiencing high job demands and a low decision latitude, job strain, and low social support show a high risk of long-term illness [51]. Consequently, presenteeism also closely associated with occupational stress [34,52]. According to a study of Boles et al., employees with high stress reported 10.2% presenteeism compared with 5.0% presenteeism among those who did not have high stress [53]. Another study suggests that with higher levels of job stress, sickness presenteeism occurred more often than sickness absence [54].

A high job demand had been identified as a risk factor for presenteeism in recent studies. Job demand leads to presenteeism and burnout [55]. Burnout has a reciprocal relationship with presenteeism since emotional exhaustion leads to presenteeism, which in turn causes more exhaustion later on [55]. Highly demanding occupations usually involve great responsibility for tasks or clients. Fewer no substitutes for workers, high workloads, and inflexible deadlines make workers feel pressure to attend. Therefore, those pressures or responsibilities lead workers with high job demands to work while sick. For example, occupations that have strong attendance demands such as medicine, nursing, and welfare and teaching occupations have a greater risk of presenteeism [56]. Similarly, difficulty in staff replacement and time pressure can be risks for presenteeism [57].

In this study, ‘lack of reward’ was defined as discontent with wages. The association of presenteeism and lack of reward can be explained by financial distress due to an unsatisfactory wage. Workers with an unsatisfactory wage, cannot easily afford to be absent from work because of their financial distress. Consequently, they are present at work even if they are sick. Along these lines, occupations with high sickness presenteeism are associated with a low monthly income [56].

The negative association between job insecurity and presenteeism may be explained by workers’ morale. Loyalty to the team and a work ethic have been mentioned as reasons to go to work ill [58,59]. However, job insecurity lowers a worker’s morale, which is associated with loyalty and a work ethic. The nature of their task, which is replaceable by a substitute, may reduce their sense of responsibility toward their duties.

Our study has some limitations. First, there is a potential mis-estimation of the experience rate of presenteeism by using a self-administered questionnaire. The participants’ answers could have been subjective. In addition, misclassification due to uncertainty of participants’ memory also can not be excluded. This misclassification can cause attenuation of statistical associations. Second, we identified associations between work-related hazardous factors and presenteeism, but could not determine the direction of causality or any temporal relationship because of the nature of a cross-sectional research design. To identify the causality, further prospective evaluation will be needed. Finally, the Cronbach’s α of high job demand and insufficient job control were not enough to expect satisfactory reliability.

In spite of these limitations, our study also has strengths and significance. First, the KWCS is the most comprehensive national survey about workers’ health and working conditions in Korea. Our study was conducted using this representative data, so the results can be considered reliable. Second, our study comprehensively analyzed work-related hazards, including workplace environmental risk exposure, long working hours, shiftwork, and occupational stress. Some studies suggesting an association between work-related factors and presenteeism have been conducted, but almost all of the studies have dealt with occupational stress; there is a lack of previous research on the potential association between presenteeism and working hours or shift work.

In general, presenteeism can cause a loss of productivity and adverse health effects [1,3,9-11]. Consequently, our results suggesting presenteeism due to long working hours, shift work, and occupational stress present an important perspective for industrial policy. We suggest that effective reductions in current long working hours and management of occupational stress can be a successful strategy for enhancing productivity and reducing presenteeism. We expect further reliable studies using objective data about workplace productivity and other adverse health effects of working hours, shift work, and occupational stress to arouse public awareness, and to provide evidence for policy making.

## Competing interests

The authors declare that they have no competing interests.

## Author’s contributions

SHJ, HCK, and SGP designed the research. SHJ and BJL performed the statistical analysis and interpreted the data. SHJ and HCK wrote the manuscript. JHL, SHM and DYJ critically revised the manuscript. All authors read and approved the final manuscript.
